# Noninvasive diagnosis of diverse gastrointestinal disorders using tantalum-rich carbon dots as an X-ray contrast agent

**DOI:** 10.1093/rb/rbag187

**Published:** 2026-09-10

**Authors:** Yibo Wei, Nan Liu, Wenjing Hou, Ran Cheng, Shaokai Sun, Gang Shu

**Affiliations:** Department of Radiology, Zhengzhou Central Hospital Affiliated to Zhengzhou University, Zhengzhou 450007, China; Department of Radiology, Tianjin Nankai Hospital, Tianjin 300100, China; Department of Diagnostic and Therapeutic Ultrasonography, Tianjin Medical University Cancer Institute and Hospital, National Clinical Research Center for Cancer, Tianjin’s Clinical Research Center for Cancer, Key Laboratory of Cancer Prevention and Therapy, Tianjin 300060, China; School of Medical Imaging, Division of Medical Technology, Tianjin Key Laboratory of Functional Imaging, Tianjin Medical University, Tianjin 300203, China; School of Medical Imaging, Division of Medical Technology, Tianjin Key Laboratory of Functional Imaging, Tianjin Medical University, Tianjin 300203, China; Department of Radiology, Tianjin Medical University Cancer Institute and Hospital, National Clinical Research Center for Cancer, Tianjin’s Clinical Research Center for Cancer, Key Laboratory of Cancer Prevention and Therapy, Tianjin 300060, China

**Keywords:** tantalum, carbon dots, contrast agent, computed tomography, gastroenterography

## Abstract

Gastrointestinal (GI) diseases pose significant diagnostic challenges due to nonspecific early symptoms and the difficulty in detecting subtle lesions. Dynamic X-ray imaging and high spatial-resolution computed tomography (CT) provide flexible and powerful tools for the precise diagnosis of diverse GI diseases. However, current clinical X-ray contrast agents (CAs) still present inherent limitations, as barium sulfate exhibits poor water solubility, and iodine-based small molecules encounter issues of iodine allergy, contraindications and generally low sensitivity. Here, we report noninvasive diagnosis of diverse GI disorders using tantalum-rich carbon dots (Ta-rCDs) as an X-ray CA. Ta-rCDs are prepared through a simple and scalable high-temperature carbonization process, featuring ultrahigh Ta content (∼48%), gram-scale yield (3.2 g per batch), ultrasmall hydrodynamic size (∼3 nm), excellent water solubility (>500 mg/mL) and strong X-ray attenuation. In models of small-intestinal stricture and esophageal leakage, Ta-rCDs enable simultaneous dynamic X-ray tracking and high-resolution CT imaging, allowing the visualization of microlesions and demonstrating efficient renal excretion upon entering the peritoneal cavity. Systematic biocompatibility assessments further confirm their good biosafety. Together these findings offer a noninvasive, real-time and high-sensitivity strategy for accurate diagnosis and monitoring of GI diseases.

## Graphical Abstract

**Graphical Abstract:**
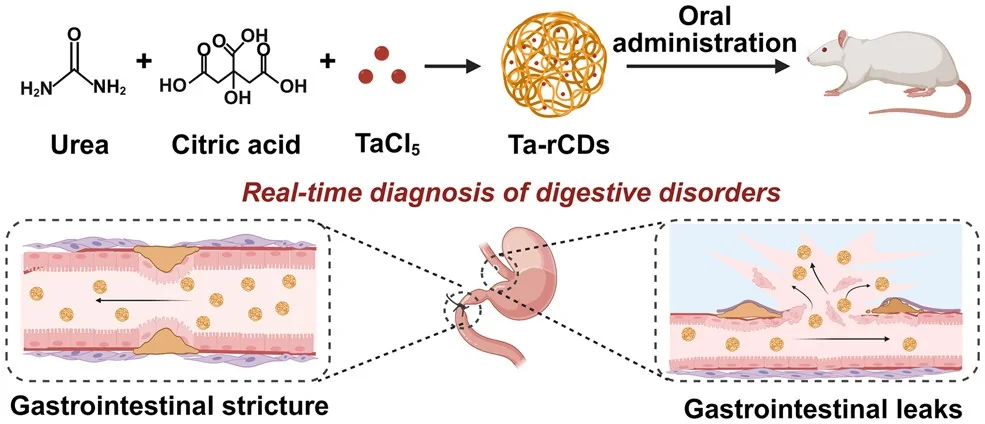

## Introduction

Gastrointestinal (GI) disorders affect millions of people worldwide, and their early symptoms are often nonspecific, leading to delayed diagnosis and an increased risk of severe complications [1–3]. Patients with a history of surgery or inflammation are particularly susceptible to subtle yet clinically significant lesions such as strictures, anastomotic leaks and perforations, many of which remain difficult to detect at an early stage [4–7]. Although endoscopy allows direct visualization of the mucosal surface, it is invasive and may cause discomfort or procedure-related injury [8, 9]. In clinical practice, X-ray fluoroscopy and computed tomography (CT) play complementary diagnostic roles. Dynamic fluoroscopy enables real-time tracking of contrast agent (CA) flow and is especially useful for identifying leakage sites, while CT provides high-resolution three-dimensional anatomical information and is well suited for detecting strictures, defining lesion extent and assessing structural abnormalities [10–12]. Together, these complementary modalities underscore the importance of oral CAs in facilitating accurate, timely and clinically meaningful evaluation of the GI tract.

However, the oral CAs currently used in clinical practice remain far from ideal. Barium sulfate (BaSO_4_) has extremely low water solubility, which limits its use to X-ray fluoroscopy and may pose safety concerns in patients with suspected obstruction or leakage [13–15]. Iodine-based small molecules provide only modest X-ray attenuation and often require high dosing to achieve satisfactory contrast, which increases the risk for patients with iodine allergy or thyroid disorders [16–18]. Their performance is suboptimal in both fluoroscopy and CT, making it difficult to obtain clear visualization of complex GI conditions. These limitations have motivated the search for new oral CAs that combine high sensitivity, excellent safety and reliable imaging performance across diverse GI scenarios [19–21].

Among various high atomic number elements [22–29], tantalum (Ta) is an attractive element for GI imaging owing to its high atomic number (73), K-edge energy (67.4 keV), chemical stability and biocompatibility [30, 31]. At clinical X-ray voltages, Ta offers superior attenuation relative to other high-Z elements [32–34]. Current Ta-based CAs, however, are typically synthesized via microemulsion, sol-gel or solvothermal methods that require complex procedures and costly precursors such as tantalum ethoxide, limiting their practical application at the high doses needed for GI imaging [35–41]. In addition, many of these agents display poor water solubility and hydrodynamic sizes above the renal filtration threshold, raising the risk of off-target accumulation in the liver or other tissues if the agents enter the peritoneal cavity or circulation through GI lesions [42, 43]. While citrate-stabilized Ta nanodots previously developed by our group demonstrated good CT performance and efficient renal clearance, their synthesis involved high-temperature hydrofluoric acid treatment [44]. These limitations highlight the need to develop Ta-based CAs with simplified fabrication, favorable safety profiles, scalable production and efficient renal clearance for GI imaging, addressing a major unmet challenge in both research and clinical practice.

Here, we developed noninvasive diagnosis of diverse GI disorders using tantalum-rich carbon dots (Ta-rCDs) as an X-ray CA (Figure 1). A high-temperature carbonization strategy was employed to prepare Ta-rCDs, providing a scalable route to ultrasmall nanoparticles (∼3 nm) with a high Ta loading (∼48%), gram-scale production (3.2 g per batch), excellent water solubility (>500 mg/mL) and strong X-ray attenuation. Following oral administration, Ta-rCDs permit dynamic X-ray tracking and high-resolution CT visualization of intestinal structure, motility and lesion localization, enabling early detection and precise characterization of microlesions in models of small intestinal stricture and esophageal leakage. Moreover, Ta-rCDs are efficiently cleared following entry into the peritoneal cavity, minimizing the risk of long-term accumulation. Comprehensive *in vitro* and *in vivo* biosafety studies further confirm their high biocompatibility, establishing Ta-rCDs as a robust platform for noninvasive, real-time and high-sensitivity imaging of GI structure and function, with considerable potential for clinical translation.

**Figure 1:**
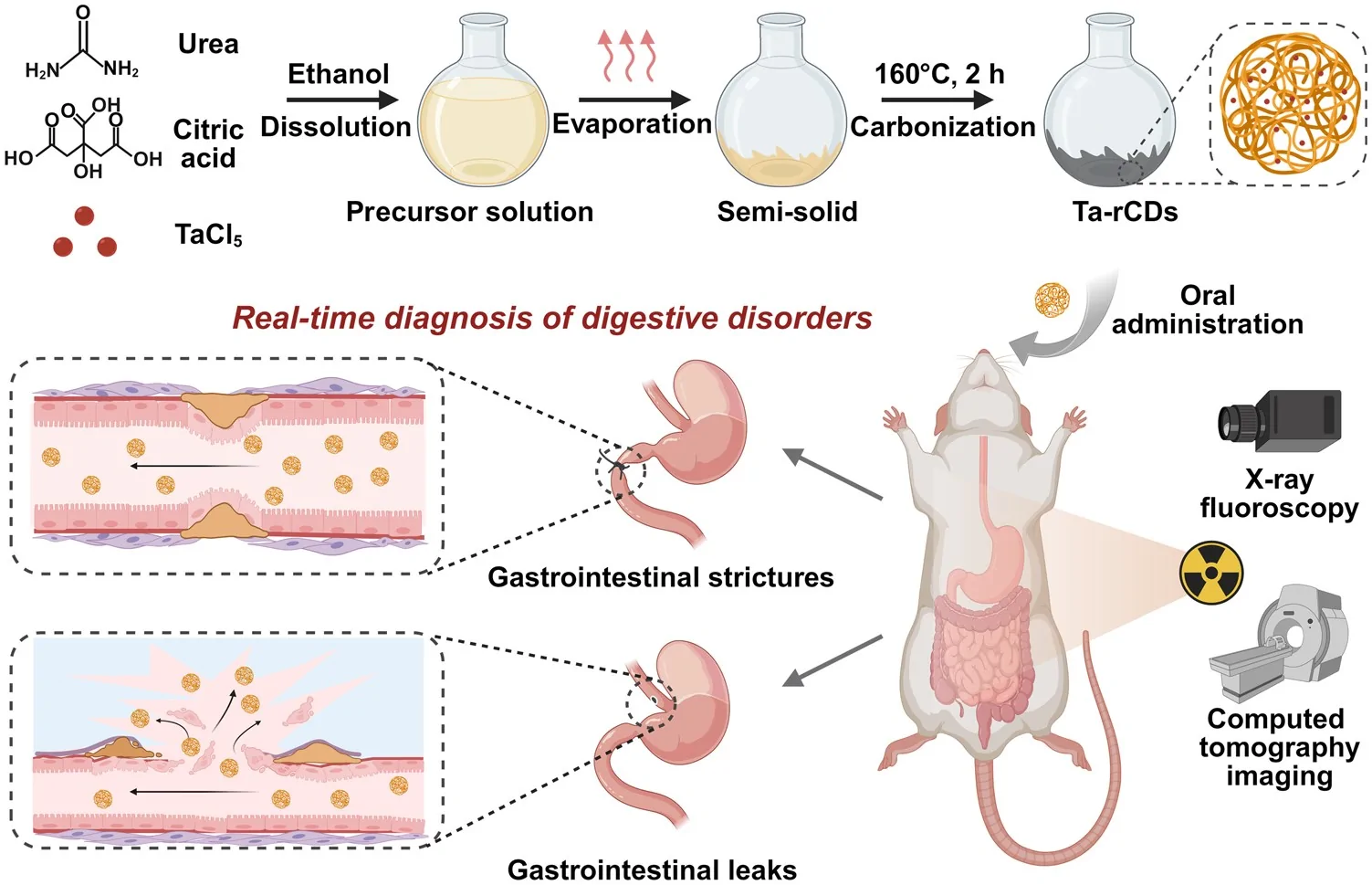
Schematic illustration of noninvasive diagnosis of diverse GI disorders using Ta-rCDs as an X-ray CA.

## Materials and methods

### Reagents and materials

Urea (99%), citric acid (CA, 99.5%) and N-methyl-D-glucamine were obtained from Shanghai Aladdin Chemical Co., Ltd. (Shanghai, China). Tantalum chloride (TaCl_5_, >98%) was purchased from Meryer Biochemical Technology Co., Ltd. (Shanghai, China). Isoflurane was obtained from RWD (Shenzhen, China). Ultrapure water was provided by Hangzhou Wahaha Group Co., Ltd. (Hangzhou, China).

### Preparation of Ta-rCDs

A Ta-containing precursor was first generated by introducing 5 g of TaCl_5_ into 62.5 mL of ethanol under constant agitation, during which a self-heating reaction accompanied by HCl release occurred, requiring operation in a ventilated enclosure. Following complete dissolution, particulate matter was removed through high-speed centrifugation (10 000 rpm, 10 min), yielding a clear solution. This filtrate was then combined with CA (2.5 g) and urea (2.5 g) in a 500 mL reaction vessel and processed by ultrasonication until optical homogeneity was achieved. The obtained homogeneous solution was concentrated under reduced pressure at 55°C to produce a viscous intermediate. This intermediate was heated at 160°C for 2 h, leading to the formation of a dark carbonaceous product containing Ta species. After cooling, the solidified product was reconstituted in deionized water (50 mL) and subjected to differential centrifugation (10 000 rpm, 10 min) to eliminate coarse aggregates. The clarified suspension was subsequently processed through membrane dialysis (2 kDa cutoff) against fresh water for 24 h with periodic solvent renewal every 6 h, followed by microfiltration (0.22 μm). The final colloidal fraction was collected after freeze-drying to afford a powder with light brown coloration.

For pH adjustment, N-methyl-D-glucamine was incorporated into the aqueous Ta-rCD dispersion to mitigate inherent acidity. A fixed compositional ratio of 5:1 (Ta-rCDs to N-methyl-D-glucamine) was adopted, producing a formulation concentration of 100 mg/mL composed of 100 mg nanomaterial and 20 mg base per milliliter of water.

### Characterization of Ta-rCDs

Transmission electron microscopy (TEM) analysis was performed using a Hitachi HT7700 system (Hitachi, Japan), while field-emission electron microscopy was conducted on a JEM-2800 instrument (JEOL, Japan). The hydrodynamic size was measured by dynamic light scattering (DLS) using a Zetasizer Nano ZS instrument (Malvern, UK). The crystal structures of CDs and Ta-rCDs were analyzed by X-ray diffraction (XRD, Rigaku D/max-2500, Cu Kα, Japan). FTIR spectra (4000–400 cm^−1^) of samples were measured on a Nicolet IS10 spectrometer using KBr as background. X-ray photoelectron spectroscopy (XPS, Axis Ultra DLD, Kratos Analytical Ltd., UK) was used to analyze the elemental composition and chemical states of Ta-rCDs. The optical absorption spectra were measured using a UV-3600 Plus UV–Vis–NIR spectrophotometer (Shimadzu, Japan), and their fluorescence emission properties were examined on an F-7000 spectrofluorometer (Hitachi, Japan). Inductively coupled plasma optical emission spectrometry (ICP-OES, Agilent 5800, USA) was used to quantify the Ta content in the obtained samples.

### Stability assessment

Ta-rCDs were dispersed in water at concentrations ranging from 10 to 500 mg/mL. Their solubility was then assessed based on visual appearance and recorded photographically. To evaluate their stability in biologically relevant media, Ta-rCDs were reconstituted at a constant concentration (20 mg/mL) in various solvents, including deionized water, normal saline (NS), phosphate-buffered saline (PBS), Dulbecco’s modified Eagle medium (DMEM) and fetal bovine serum (FBS). The resulting suspensions were maintained at 4°C, and their physical state was monitored over time. Any signs of precipitation or aggregation were recorded on Days 0, 7, 14 and 30 to assess long-term colloidal stability.

To evaluate the stability of Ta-rCDs in the GI environment, the dispersion behavior and Ta^5+^ release were investigated in simulated GI fluids. Ta-rCDs (20 mg/mL) were sealed in dialysis bags (MWCO 500 Da) and immersed in simulated gastric fluid (SGF, pH 1.2) and simulated intestinal fluid (SIF, pH 6.8), respectively, and incubated at 37°C with continuous shaking. At predetermined time points, the dialysate was collected. The cumulative release of Ta^5+^ was quantified by ICP-OES.

### *In vitro* CT imaging evaluation

CT imaging ability of Ta-rCDs *in vitro* was evaluated using a clinical dual-energy CT platform (Somatom Drive, Siemens Healthineers, Erlangen, Germany). A series of Ta-containing aqueous dispersions (0–200 mM Ta) were prepared for imaging analysis. All scans were performed under clinical acquisition conditions with a field of view of 256 × 256 mm, a slice thickness of 0.5 mm and tube voltage settings ranging from 70 to 140 kV with adaptive current modulation. The corresponding CT attenuation values (Hounsfield units, HU) were quantified from the generated images at different voltages. For comparison, iohexol solutions and BaSO_4_ suspensions containing equivalent concentrations of radiopaque elements were analyzed under identical imaging parameters to benchmark the X-ray attenuation capability of Ta-rCDs.

### Animal models

Animal experiments were approved by the Animal Care and Use Committee of Tianjin Medical University (approval No. TMUaMEC 2024012) and carried out in accordance with institutional ethical guidelines. Male Sprague–Dawley (SD) rats (220–250 g) were supplied by SPF Biotechnology Co., Ltd. (Beijing, China).

### *In vivo* GI tract imaging using CT and X-ray

Healthy rats were fasted for 24 h prior to imaging while being allowed free access to water. The rats were anesthetized and orally administered with either Ta-rCDs or iohexol (6 mL, 0.1 M Ta or I, *n* = 3 biologically independent samples). *In vivo* GI CT imaging was subsequently conducted under the same acquisition conditions as those used for the *in vitro* CT experiments. Longitudinal imaging was carried out from pre-administration to 48 h post-gavage (5, 10, 30 min, 1, 2, 24 and 48 h) and reconstructed datasets were used to evaluate GI distribution over time.

For X-ray imaging, the rats were similarly anesthetized and administered Ta-rCDs, iohexol or BaSO_4_ (2 mL, 0.5 M Ta, iohexol [I] or barium [Ba], *n* = 3 biologically independent samples) via oral gavage. The GI tract was serially imaged by X-ray at multiple time points spanning pre-administration to 48 h post-gavage using a Shimadzu 500MA digital imaging system (Shimadzu, Japan), with a tube voltage of 50 kV and adaptive current modulation.

### Small intestinal stricture model and CT imaging

A 24 h fasting protocol was applied to healthy rats before surgery, while water was available *ad libitum*. Following anesthesia, abdominal hair was shaved, and a midline laparotomy was performed to expose the abdominal cavity. The small intestine was gently exteriorized to identify the pylorus, after which a partial ligation was created at the proximal duodenum approximately 1 cm distal to the pylorus using a silk suture, leaving a narrow lumen to establish a reversible intestinal stricture. The intestines were carefully returned to the abdominal cavity, followed by layered closure of the abdominal wall.

After recovery, rats received an oral dose of Ta-rCDs solution (6 mL, 0.1 M Ta), followed by serial CT imaging at multiple time points to identify the stricture location and evaluate luminal obstruction (*n* = 3 biologically independent samples). Subsequently, the ligature was released to re-establish intestinal continuity, and a second round of CT imaging was performed to confirm successful recanalization and monitor the passage of the CA through the intestinal tract. At the conclusion of the study, the animals were euthanized and *ex vivo* CT scans of the excised GI tracts were obtained for further structural verification.

### Esophageal leakage model and CT/X-ray imaging

Healthy rats underwent a 24 h fasting period before the procedure, with free access to water. After inducing anesthesia, the abdomen was shaved and sterilized. The abdominal cavity was accessed via a midline incision, followed by careful dissection and elevation of the distal esophagus at the gastroesophageal junction. Esophageal leakage was modeled by transverse incision of about 50% of the left lateral esophageal wall, causing mucosal rupture. Hemostasis was achieved by gently pressing the wound with a sterile cotton swab, and the absence of active bleeding was confirmed before repositioning the organs and closing the abdominal cavity.

Following model establishment, Ta-rCDs, iohexol or BaSO_4_ were administered via a catheter (2 mL, 0.5 M Ta, I or Ba), and X-ray imaging was subsequently conducted in a time-resolved manner (0–2 h post-administration) to visualize GI distribution. Animals were then euthanized with an overdose of anesthetic gas, after which the esophagus and stomach were collected for *ex vivo* photographic analysis.

Following catheter administration of Ta-rCDs (2 mL, 0.1 M), CT imaging was performed at predefined time points. *In vivo* and *ex vivo* X-ray imaging of the esophagus and stomach was then conducted. To evaluate the systemic exposure and biosafety of the leaked CA, a separate cohort of rats (*n* = 3 biologically independent samples) received the same dose of Ta-rCDs (2 mL, 0.5 M) via the catheter. At selected time points after administration, samples of blood and urine, as well as key organs including heart, liver, spleen, lung and kidney, were collected. Ta concentrations in these samples were quantified by ICP-OES. For biochemical assessment, blood samples were collected before and at 12 h after leakage, and serum samples were obtained and used for biochemical analysis of hepatic and renal function.

### Cell culture

The 3T3-L1 mouse embryonic fibroblast cell line was obtained from the Cell Bank of the Chinese Academy of Sciences (Shanghai, China). NCM460 human normal colonic epithelial cells and RBE4 rat brain microvascular endothelial cells were purchased from Otwo Biotech Inc. (Shenzhen, China). All cell lines were maintained under standard culture conditions in DMEM supplemented with 5% FBS and 1% penicillin–streptomycin. Cells were incubated in a humidified atmosphere at 37°C with 5% CO_2_.

### *In vitro* cytotoxicity assessment

Cell viability following exposure to Ta-rCDs was quantified using a 3-(4,5-dimethylthiazol-2-yl)-2,5-diphenyltetrazolium bromide (MTT)-based colorimetric assay in 3T3-L1, NCM460 and RBE4 cells. Cells were seeded into 96-well plates (1 × 10^5^ cells per well, 200 μL complete medium) and incubated for 24 h to allow adhesion. After attachment, the culture medium was replaced with fresh medium containing graded concentrations of Ta-rCDs (0-3000 mg·L^−1^), and cells were further incubated for 24 h. Metabolic activity was then assessed by adding MTT solution (5 mg·mL^−1^, 10 μL per well) and incubating for 4 h to enable intracellular reduction of MTT into formazan crystals. Following incubation, the supernatant was removed, and formazan crystals were dissolved in dimethyl sulfoxide (DMSO) (120 μL per well). Absorbance was recorded at 490 nm using a microplate reader, and cell viability was calculated relative to untreated controls.

### Hemocompatibility assessment

Fresh blood from healthy rats was collected via the orbital sinus into EDTA-coated tubes to prevent coagulation. Erythrocytes were subsequently separated by centrifugation (3000 rpm, 5 min), washed three times with NS and resuspended in NS.

For hemolysis evaluation, red blood cell (RBC) suspensions were exposed to Ta-rCDs at different concentrations (0.5–50 mg·mL^−1^). Distinct control conditions were established, including isotonic saline as the negative control and deionized water as the positive control. All samples were incubated at 37°C for 4 h, followed by centrifugation (3000 rpm, 5 min) to pellet intact erythrocytes. The supernatants were collected, and hemoglobin release was quantified by measuring absorbance at 541 nm after transfer to a 96-well plate. The hemolysis ratio of each Ta-rCDs concentration was calculated relative to the negative and positive controls (*n* = 3 independent experiments), and visual inspection of the supernatants was performed to confirm erythrocyte integrity.


$$\text{Hemolysis}\;\text{rate}\;(\%)=\frac{A-A_{\text{negative}}\;}{A_{\text{positive}}-A_{\text{negative}}}\times 100\%.$$

### *In vivo* toxicity evaluation

The systemic biocompatibility of Ta-rCDs following oral administration was assessed in rats using a combination of physiological, biochemical and histological analyses. The rats (∼200 g) were gavaged with 2 mL of Ta-rCDs solution (0.5 M, corresponding to 5 mmol Ta/kg body weight) or NS (*n* = 3 biologically independent samples). General health status was monitored by recording body weight every two days over a 14-day observation period. Peripheral blood was collected from the tail vein at predefined time points (0, 1, 7 and 14 days post-administration), followed by serum isolation for biochemical analysis including liver and kidney function. At designated time points post-administration, rats were euthanized for sample collection. The GI tract (esophagus, small intestine and large intestine), major organs (heart, liver, spleen, lung and kidney) and ocular tissues (cornea, retina and optic nerve) were harvested for histopathological examination. To assess excretion and systemic exposure of Ta-rCDs, Ta concentrations in feces, urine and blood were analyzed by ICP-OES following time-resolved collection.

### Statistical analysis

Data were statistically analyzed and visualized, and are expressed as mean ± standard deviation (s.d.).

## Results and discussion

### Synthesis and characterization of Ta-rCDs

TaCl_5_, CA and urea were employed as precursors for the synthesis of Ta-rCDs via carbonization. Given the pronounced hydrolytic susceptibility of Ta^5+^ species, anhydrous ethanol was employed as a coordinating medium to suppress premature hydrolysis and ensure homogeneous molecular-level dispersion. After gentle solvent removal, the obtained viscous intermediate was thermally carbonized under controlled conditions to yield uniform Ta-rCDs.

Optimization of precursor composition and reaction parameters led to improved yield and colloidal uniformity, with the best performance achieved at a CA:urea:TaCl_5_ mass ratio of 1:1:2 and a pyrolysis temperature of 160°C for 2 h. This route affords gram-scale Ta-rCDs production (∼3.2 g per batch, Supplementary Figure S1) under mild conditions.

Ta-rCDs exhibited a uniform spherical morphology under TEM, with an average core diameter of approximately 2 nm (Figure 2A). HRTEM images revealed clear lattice fringes with a spacing of about 0.2 nm, assigned to the (100) plane of graphitic carbon (Figure 2B) [45, 46]. A narrow hydrodynamic size distribution of approximately 3 nm was observed by DLS in H_2_O, NS and PBS (Figure 2C). Such an ultrasmall size is favorable for safe metabolism and efficient renal clearance, minimizing long-term retention risks [47, 48]. Besides, XRD analysis indicated that both pristine CDs and Ta-rCDs exhibited predominantly amorphous structures, as reflected by broad and weak diffraction features. A wide peak located near 26° was identified in both samples, which can be ascribed to the (002) interlayer spacing of graphitic carbon [49], indicative of limited short-range structural ordering. In the case of Ta-rCDs, an additional diffuse reflection appeared around 53°, suggesting that Ta incorporation may induce local lattice strain (Supplementary Figure S2).

**Figure 2:**
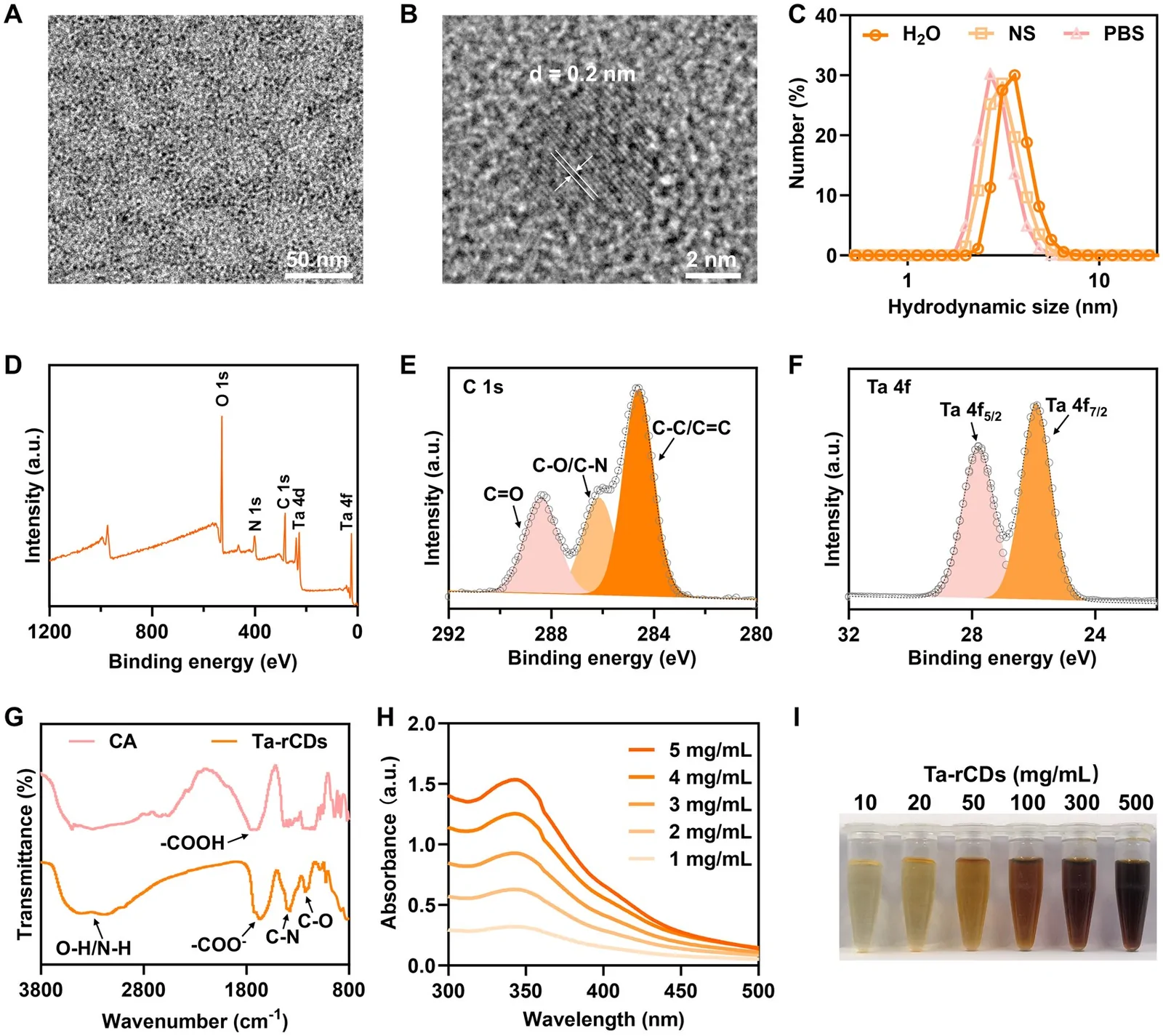
Synthesis and characterization of Ta-rCDs. (**A**) TEM image of Ta-rCDs. (**B**) HRTEM image of Ta-rCDs. (**C**) Hydrodynamic sizes of Ta-rCDs in H_2_O, NS and PBS. (**D**) XPS survey spectra of Ta-rCDs. High-resolution XPS spectra of C 1s (**E**) and Ta 4f (**F**). (**G**) FTIR spectra of CA and Ta-rCDs. (**H**) UV–Vis–NIR absorption spectra of Ta-rCDs at different concentrations in aqueous solution. (**I**) Photographs of Ta-rCDs aqueous dispersions with increasing concentrations (10–500 mg/mL).

XPS was employed to clarify the surface composition and electronic structure of Ta-rCDs. The survey spectrum evidenced the coexistence of C, O, N and Ta, supporting the successful integration of Ta species into the carbonaceous framework (Figure 2D). Further analysis of the high-resolution spectra suggested a chemically heterogeneous surface with abundant functionalization. The C 1s profile reflected a carbon backbone enriched with C-C/C = C, C-O/C-N and C = O bonds (Figure 2E). In parallel, the Ta-related signals were characteristic of Ta^5+^ species, indicating that Ta predominantly exists in a highly oxidized state within the hybrid structure (Figure 2F). Contributions from O- and N-containing environments further confirmed surface functionalization and heteroatom incorporation, consistent with partial graphitization of the carbon matrix. A weak feature overlapping in the high binding energy region was attributed to the Ta 4p_3/2_ signal (Supplementary Figure S3), without affecting the overall chemical assignment [50–52]. Collectively, these observations reveal that Ta-rCDs possess a heteroatom-enriched surface with abundant oxygen- and nitrogen-functional groups, which support their hydrophilicity and potential for chemical modification.

Fourier transform infrared spectroscopy (FTIR) was used to probe the coordination structure of Ta-rCDs. The FTIR spectrum of Ta-rCDs exhibited characteristic vibrations associated with abundant surface functional groups. Broad bands centered at 3412 cm^−1^ and 3200 cm^−1^ were attributed to O-H and N-H stretching modes, respectively, reflecting extensive hydrogen-bonding interactions on the surface. A signal at 1380 cm^−1^ was were linked to C-N stretching within aromatic amine structures, suggesting successful nitrogen incorporation into the carbon framework. In addition, the absorption feature at 1220 cm^−1^ was associated with C-O stretching vibrations, consistent with carboxyl groups originating from citric acid. Notably, the characteristic carboxyl absorption shifted from 1710 cm^−1^ in CA to 1670 cm^−1^ in Ta-rCDs, suggesting chelation between the carboxyl groups and Ta^5+^ (Figure 2G) [53–55]. The optical absorption behavior of Ta-rCDs was evaluated using UV–Vis–NIR spectroscopy. A distinct absorption band centered at 340 nm was observed, which is typically associated with n-π* transitions of C = O and C–N bonds. This feature suggests the presence of abundant oxygen- and nitrogen-containing surface functionalities within the carbon framework (Figure 2H) [56]. Fluorescence emission spectra further demonstrated wavelength-dependent emission behavior with a maximum excitation wavelength at 340 nm, resulting in bright blue fluorescence under ultraviolet irradiation (Supplementary Figure S4) [55, 57]. The above optical findings are consistent with the structural characterizations and collectively confirm the successful synthesis of Ta-rCDs. Additionally, ICP-OES measurements quantitatively determined that Ta accounts for approximately 48% of the Ta-rCDs. Collectively, these results verify the successful formation of Ta-rCDs with effective Ta incorporation, highlighting their suitability for imaging applications.

High water solubility and robust colloidal stability are essential characteristics for high-performance CT imaging probes. The Ta-rCDs could form a clear solution even at a concentration as high as 500 mg/mL without observable aggregation, demonstrating their exceptional solubility (Figure 2I). The long-term stability assessment showed that no precipitation or aggregation was detected during this period, indicating that Ta-rCDs maintain excellent dispersion across diverse physiological environments, providing a reliable foundation for *in vivo* applications at high-concentration (Supplementary Figure S5). To evaluate Ta^5+^ release in the GI environment, Ta-rCDs were incubated in SGF (pH 1.2) and SIF (pH 6.8), respectively. No visible precipitation was observed during the incubation period, and the cumulative release of Ta^5+^ quantified by ICP-OES was below 2% of the total Ta content in both fluids with no significant time-dependent increase, confirming their biosafety for oral administration (Supplementary Figure S6).

### *In vitro* CT imaging performance

The *in vitro* CT imaging performance of Ta-rCDs was evaluated using a clinical dual-energy CT (DECT) system. Comparative CT imaging was performed for iohexol, BaSO_4_ and Ta-rCDs at concentrations ranging from 0 to 200 mM (I, Ba or Ta) under tube voltages of 70–140 kV. HU values were measured to quantify image contrast and signal intensity. A linear correlation between CT values and concentration was observed for iohexol, BaSO_4_ and Ta-rCDs (Figure 3 and Supplementary Figure S7), indicating good concentration-dependent imaging capability. However, their responses to tube voltage differed markedly. The CT signals of iohexol and BaSO4 decreased rapidly with increasing voltage. In contrast, Ta-rCDs exhibited slightly lower signals than iohexol below 80 kV but surpassed it above 80 kV, and showed lower signals than BaSO_4_ below 90 kV but exceeded it above 90 kV, while maintaining relatively stable signals across higher voltage ranges (Supplementary Figure S8). This distinct energy response can be attributed to the difference in K-edge energies among Ta (67 keV), Ba (37 keV) and iodine (33 keV), where the higher K-edge of Ta enables more efficient photon absorption and improved signal retention under high-energy X-rays. These findings highlight the excellent energy response characteristics of Ta-rCDs and suggest their suitability for CT imaging within clinically relevant voltage ranges.

**Figure 3:**
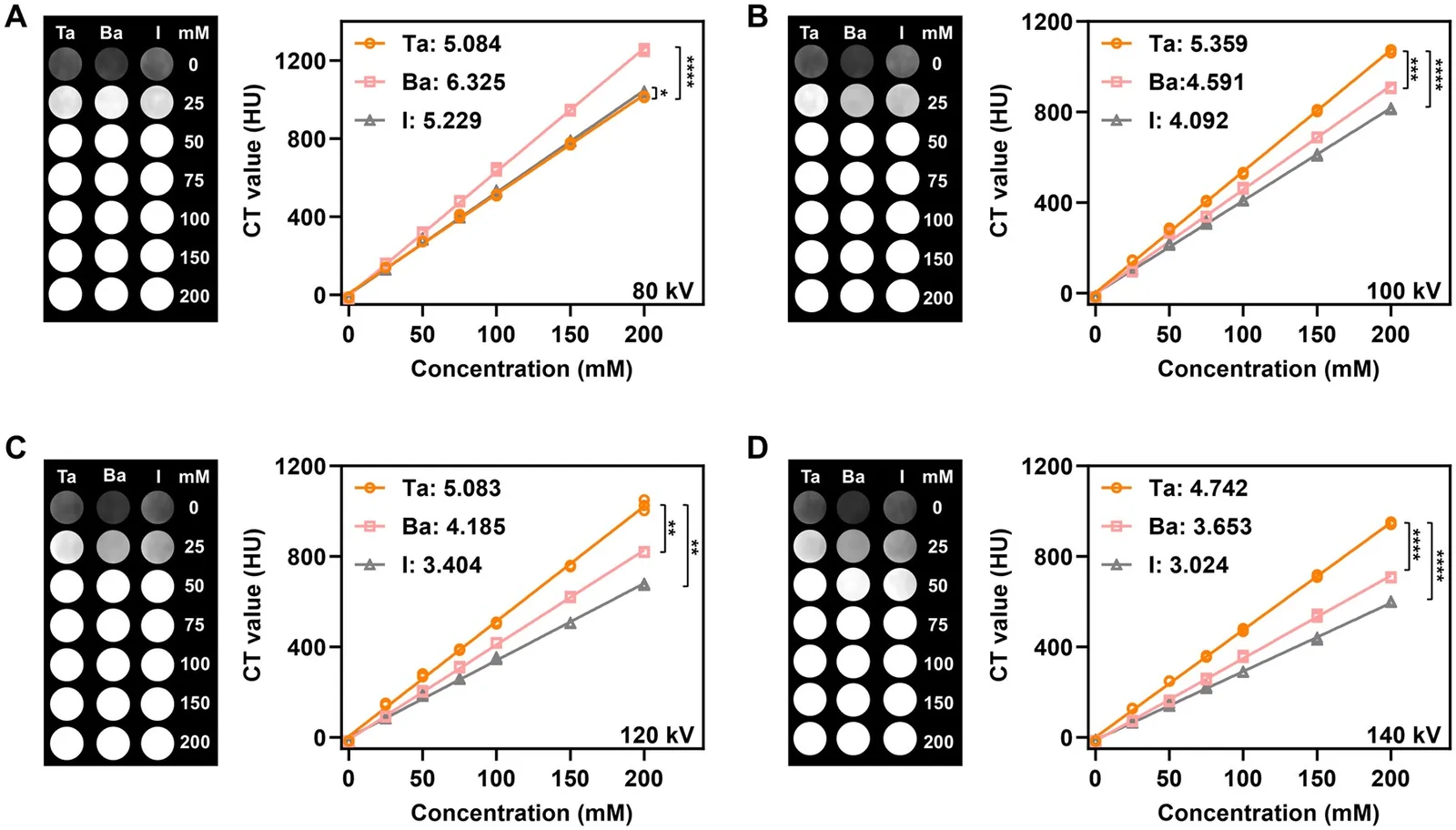
*In vitro* CT imaging of Ta-rCDs. Conventional CT images (left) and CT signal curves (right) of Ta-rCDs, iohexol and BaSO_4_ at different concentrations (0, 25, 50, 75, 100, 150 and 200 mM Ba, I or Ta) under different tube voltages of 80 kV (**A**), 100 kV (**B**), 120 kV (**C**) and 140 kV (**D**). WW = 250 HU, WL = 50 HU. Data are presented as mean ± s.d. (*n* = 3 independent experiments). Statistical analysis was performed using two-way ANOVA followed by Tukey’s *post hoc* test. ****P < 0.0001, *** *P *< 0.001, ** *P *< 0.01, * *P *< 0.05; ns, not significant.

### *In vivo* GI imaging using CT and X-ray

Accurate evaluation of GI diseases relies on imaging techniques that provide both structural resolution and functional information. X-ray fluoroscopy enables real-time observation of GI dynamics, while CT imaging offers high-resolution anatomical and three-dimensional structural information. Together, these modalities provide complementary insights into GI morphology and physiology. In this context, the strong X-ray attenuation of Ta-rCDs prompted further investigation of their suitability as GI CT CAs.

To this end, the GI tract visualization ability of Ta-rCDs was assessed in comparison with the clinically used iohexol. Ta-rCDs or iohexol (6 mL, 0.1 M Ta or I) were orally administered to healthy SD rats, and multiphase CT imaging was subsequently performed. Within 10 min after administration, volume-rendered (VR) CT imaging showed rapid accumulation of Ta-rCDs in the stomach and small intestine, producing strong contrast that clearly delineated the gastric wall and intestinal lumen. Over the following 20 min, the CAs advanced progressively through the small intestine, reaching its most distinct visualization at 30 min. Two hours after administration, the probe reached the cecum and colon, clearly outlining the morphology and contour of the lower GI tract. At 24 h, most Ta-rCDs were excreted, and by 48 h, they were almost completely cleared from the body. In contrast, although iohexol also produced enhancement in the stomach and small intestine, it was difficult to delineate the GI contour effectively (Figure 4A). Further quantitative analysis demonstrated that across all imaging time points, the CT attenuation values of GI segments in the Ta-rCDs group were consistently higher than those in the iohexol group (Figure 4B–D). Overall, Ta-rCDs demonstrated superior performance to iohexol in CT-based GI imaging, providing higher X-ray attenuation, more sustained contrast and effective visualization even at lower concentrations.

**Figure 4:**
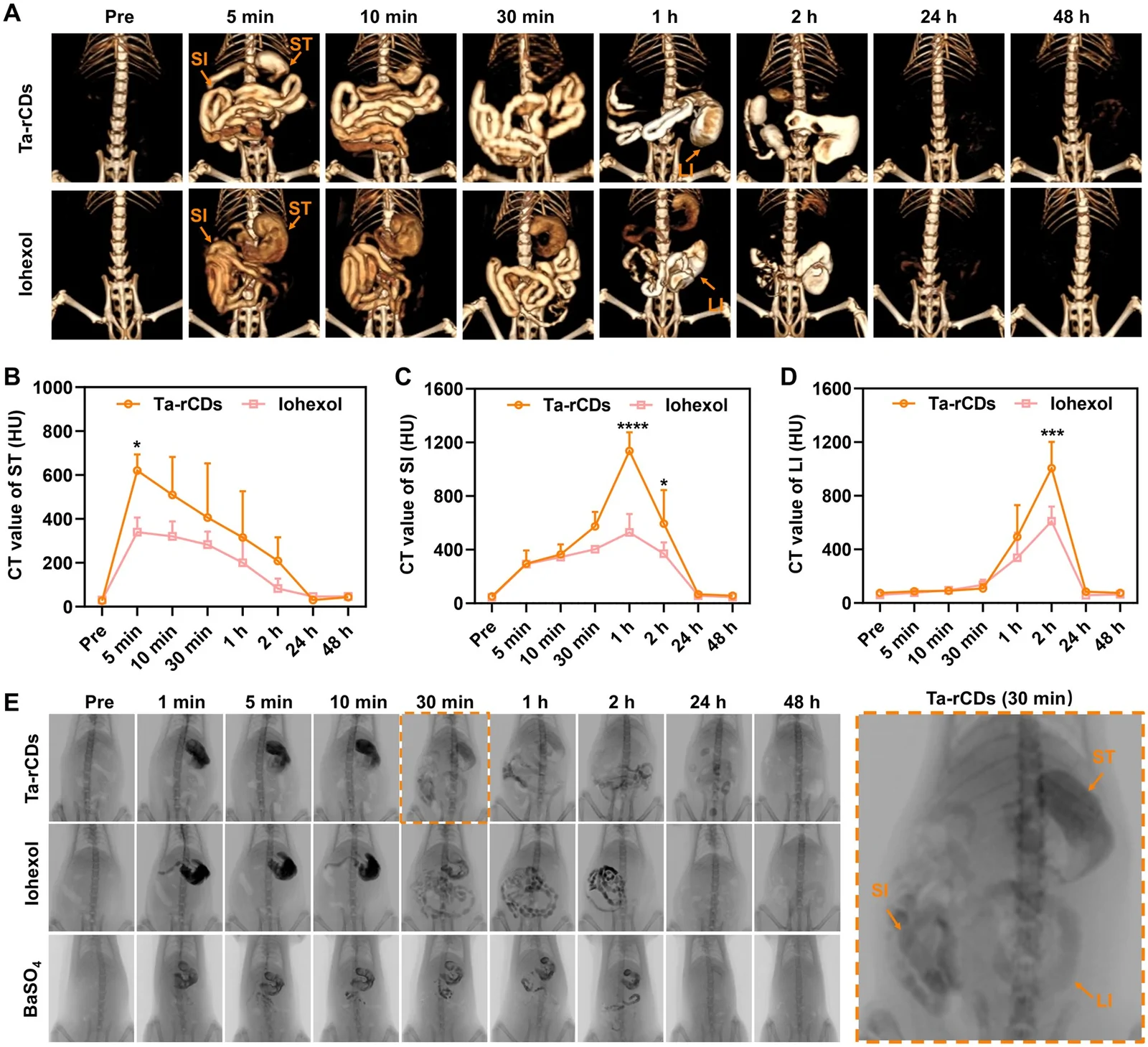
*In vivo* GI imaging using CT and X-ray. (**A**) Three-dimensional VR CT reconstruction of the GI tract following oral administration of Ta-rCDs or iohexol (6 mL, 0.1 M Ta or I). Quantitative CT attenuation analysis in the stomach (**B**), small intestine (**C**) and large intestine (**D**) at multiple post-administration time points for both CAs. (**E**) Real-time X-ray fluoroscopic imaging of the GI tract after oral delivery of Ta-rCDs, iohexol or BaSO_4_ (2 mL, 0.5 M Ta, I or Ba). Data are presented as mean ± s.d. (*n* = 3 biologically independent samples). Statistical analysis was performed using a nonparametric two-way ANOVA followed by Šidák’s *post hoc* test. ****P < 0.0001, ***P < 0.001, **P < 0.01, *P < 0.05; ns, not significant.

To further validate their dynamic imaging capability, Ta-rCDs were evaluated using a clinical GI X-ray system. Upon oral administration (2 mL, 0.5 M Ta, Ba or I), the stomach exhibited pronounced contrast enhancement, enabling distinct delineation of its contour. As time progressed, Ta-rCDs gradually transited into the small intestine, where the intestinal outline was clearly captured. By 2 h post-administration, most of the CAs had emptied from the stomach and accumulated in the cecum and large intestine. At 48 h, nearly all Ta-rCDs were excreted. Rats in iohexol group provided effective GI contrast comparable to that of Ta-rCDs group (Figure 4E), but its use can be limited by high-dose requirements and iodine-induced hypersensitivity. Conversely, BaSO_4_ afforded considerably weaker and more heterogeneous GI contrast, manifesting as overall lower signal intensity with patchy distribution. Such suboptimal performance presumably stems from its poor aqueous solubility, which results in non-homogeneous dispersion and localized aggregation, in stark contrast to the well-dispersed Ta-rCDs and iohexol. However, Ta-rCDs offer a comparable imaging option while minimizing these risks, serving as a biocompatible, iodine-free alternative. Additionally, real-time X-ray fluoroscopic imaging revealed the dynamic transit of Ta-rCDs through the esophagus, stomach and duodenum, allowing continuous observation of GI motility and function (Movie 1).

Collectively, Ta-rCDs enable both sustained CT contrast and real-time fluoroscopic imaging of the GI tract for structural and functional assessment. This dual-modality capability positions Ta-rCDs as a promising next-generation imaging platform for precise diagnosis and dynamic monitoring of GI diseases.

### Real-time imaging and monitoring of GI stricture and leakage using Ta-rCDs

A rat model of small intestinal stricture was established to assess the performance of Ta-rCDs in GI imaging. After oral administration of Ta-rCDs (6 mL, 0.1 M Ta), multiphase CT imaging was conducted to identify the stricture site and track postoperative recovery (Figure 5A). In the sham group, oral administration of Ta-rCDs resulted in uniform and homogeneous opacification of the entire small intestine at all observed time points, with smooth and continuous luminal filling without any filling defects or abrupt narrowing (Supplementary Figure S9). In contrast, CT imaging acquired at 5- and 10-min post-administration in the stricture group revealed localized filling defects in the intestinal lumen corresponding to stricture sites, accurately delineating the areas of stricture. Dissection and *ex vivo* CT imaging of the corresponding intestinal segments confirmed high spatial consistency with *in vivo* observations, validating the accuracy of Ta-rCDs-enhanced CT (Supplementary Figure S10). Following ligation release, CT imaging at 20 min demonstrated significant restoration of lumen patency (Figure 5B). Curved planar reconstruction (CPR) images acquired at 10 and 20 min further illustrated the dynamic transition from stricture to recovery, highlighting the capacity of Ta-rCDs to provide real-time visualization of treatment responses (Figure 5C). Moreover, *ex vivo* photographs and CT images of the dissected intestinal segments further confirmed the recovery of the stricture, corroborating the *in vivo* imaging findings (Figure 5D).

**Figure 5:**
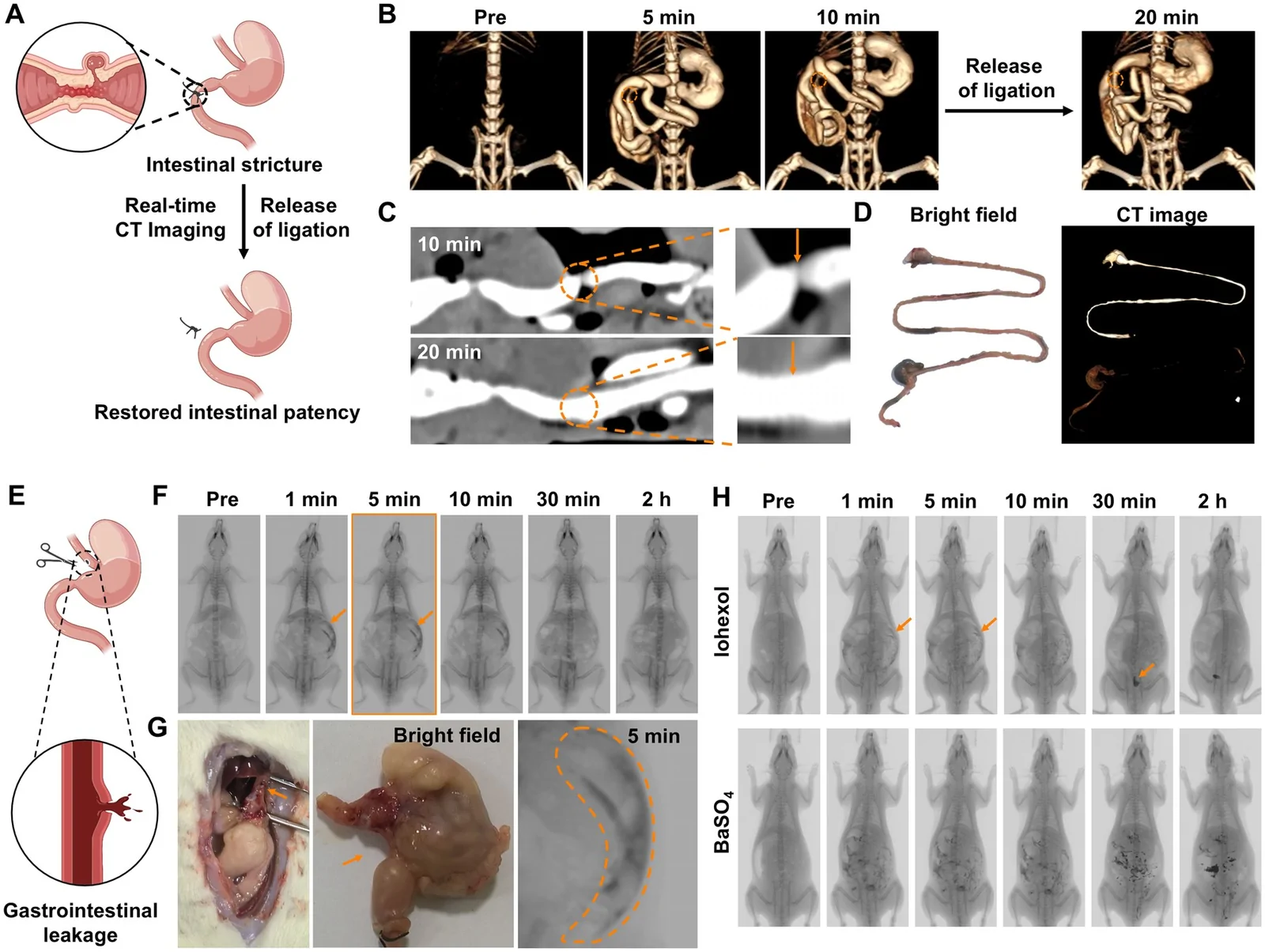
CT and X-ray diagnosis of GI stricture and leakage. (**A**) Schematic representation of the surgically induced small intestinal stricture model. (**B**) Three-dimensional VR CT images acquired at different time points before and after surgical release of the stricture following oral administration of Ta-rCDs. (**C**) CPR images of the small intestine obtained at 10 min (pre-release) and 20 min (post-release). WW = 400 HU, WL = 60 HU. (**D**) *Ex vivo* photographs and CT imaging of small intestines at 20 min post-administration of Ta-rCDs. (**E**) Schematic of the surgically induced esophageal leakage model. (**F**) Esophageal leakage was visualized in real time by X-ray fluoroscopy after oral administration of Ta-rCDs (2 mL, 0.5 M Ta). (**G**) *Ex vivo* imaging of the esophagus and stomach revealed the leakage site located at the lower esophageal wall. (**H**) Comparable fluoroscopic imaging was performed using iohexol or BaSO_4_ under identical conditions (2 mL, 0.5 M I or Ba).

Early-stage GI anastomotic leakage is often clinically silent, making it a frequent postoperative complication that is difficult to identify. Delayed detection can result in severe outcomes, including peritonitis, abscess formation or sepsis. To explore the diagnostic potential of Ta-rCDs for detecting GI leakage, a lower esophageal anastomotic leakage model was employed (Figure 5E). In the sham group, oral administration of Ta-rCDs (0.5 M, 2 mL) resulted in no extravasation of CA outside the lumen at any time point, confirming the integrity of the esophageal structure (Supplementary Figure S11). In contrast, rats in leakage model group were orally administered Ta-rCDs (2 mL, 0.5 M) for dynamic X-ray fluoroscopy. Real-time imaging showed rapid extravasation of Ta-rCDs from the rupture site, forming patchy high-density shadows that extended into the abdominal cavity, indicative of acute leakage (Movie 2). The intensity of the leaked contrast gradually decreased and almost disappeared within 30 min, demonstrating minimal residual retention and rapid *in vivo* clearance (Figure 5F). *Ex vivo* imaging revealed a discontinuity in the lower esophageal wall consistent with X-ray observations (Figure 5G). For comparison, iohexol also enabled clear visualization of the leakage site and contrast extravasation, with signal intensity comparable to that of Ta-rCDs. In contrast, BaSO_4_ extravasation appeared as scattered, heterogeneous dense opacities throughout the abdominal cavity, with no obvious clearance over time, likely due to its poor solubility and non-biodegradable nature, severely limiting its usefulness for leakage detection (Figure 5H). The leakage site was clearly visualized in *ex vivo* images of the esophagus and stomach, consistent with *in vivo* findings (Supplementary Figure S12).

To further investigate the biodistribution and clearance of leaked Ta-rCDs, an additional oral administration (6 mL, 0.1 M) was performed, followed by CT imaging at multiple time points. The results showed patchy high-density shadows on one side of the abdominal cavity, with a small amount of Ta-rCDs possibly dispersing to the opposite side approximately 30 min after administration. Concurrent bladder enhancement indicated absorption of the Ta-rCDs through the peritoneum followed by excretion via the urinary system. By around 2 h post-administration, most of the Ta-rCDs in the abdominal cavity were no longer visible, suggesting that the majority had been absorbed and eliminated (Supplementary Figure S13). To assess the systemic exposure and biosafety of the leaked Ta-rCDs, Ta concentrations in blood, urine and major organs (heart, liver, spleen, lung and kidney) were quantified by ICP-OES at designated time points after administration and serum biochemical parameters were measured before leakage and 12 h thereafter. Ta concentrations in blood and urine peaked at approximately 2 h and returned to baseline within 12 h. In major organs, Ta levels reached their maximum at 4 h and similarly declined to baseline by 12 h, indicating efficient systemic clearance (Supplementary Figure S14). Moreover, serum biochemical indices, including liver and kidney function markers, remained comparable before and after leakage, suggesting that the leaked Ta-rCDs caused no detectable acute hepatotoxicity or nephrotoxicity (Supplementary Figure S15).

To further evaluate the local biosafety of Ta-rCDs, H&E staining of the esophagus and small intestine was performed in normal rats as well as in rat models of intestinal stricture and GI leakage before and 24 h after Ta-rCDs administration (Supplementary Figure S16). In normal rats, the GI tissues exhibited intact histological architecture without evidence of inflammation or tissue injury. As expected, mild localized inflammatory cell infiltration was observed at the lesion sites in the stricture and leakage models prior to administration, reflecting the underlying pathological conditions. Notably, no additional histopathological abnormalities, including epithelial disruption, tissue injury or aggravated inflammatory infiltration, were detected 24 h after Ta-rCDs administration in any group. These findings demonstrated that Ta-rCDs exhibited excellent local biocompatibility and did not exacerbate pre-existing GI lesions.

These above results demonstrated that Ta-rCDs enable high-resolution CT imaging and real-time fluoroscopic tracking of GI leakage, combining the anatomical detail of CT with the dynamic tracking capability of X-ray, while their renal clearance and favorable biodistribution indicate good biocompatibility and safety.

### Biosafety assessment

*In vitro* and *in vivo* studies were performed to assess the biosafety profile of Ta-rCDs. The *in vitro* biosafety of Ta-rCDs was first assessed by co-culturing various concentrations with 3T3-L1, NCM460 and RBE4 cells. At a concentration as high as 3000 mg/L, more than 80% of cells remained viable, suggesting good cytocompatibility of Ta-rCDs (Figure 6A and Supplementary Figure S17). Importantly, hemolysis remained below 5% at all tested concentrations, demonstrating excellent blood compatibility (Supplementary Figure S18).

**Figure 6:**
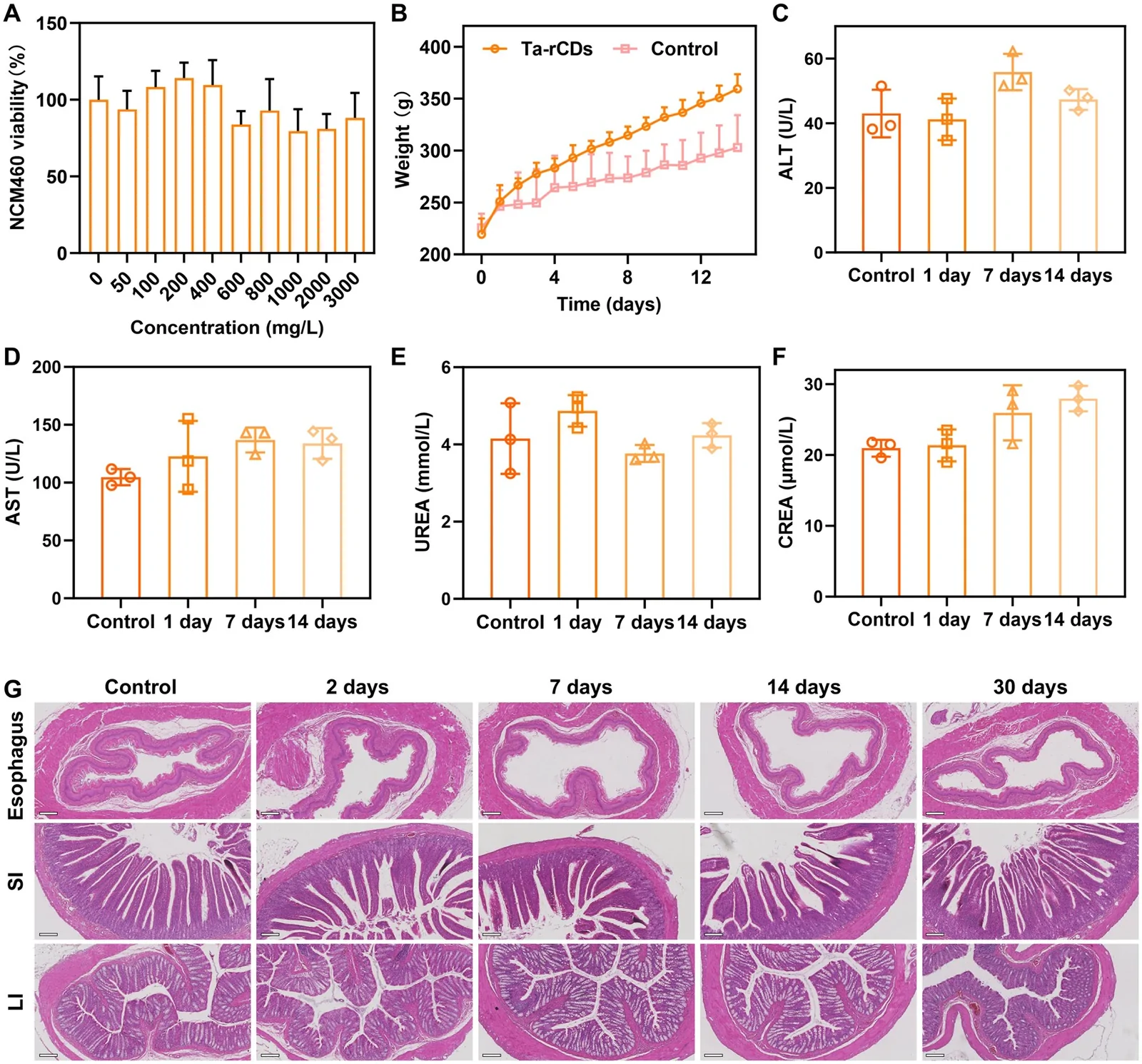
*In vitro* and *in vivo* biosafety assessment. (**A**) Cell viability of NCM460 cells after incubation with different concentrations of Ta-rCDs (0–3000 mg/L). Data are presented as mean ± SD (*n* = 6 independent experiments). (**B**) Body weight variations of rats over 14 days following oral administration of Ta-rCDs (5 mmol Ta/kg), in comparison with the control group. Biochemical analyses of rats on Days 0, 1, 7 and 14 after oral administration of Ta-rCDs, showing changes in liver function indicators ALT (**C**) and AST (**D**) and kidney function indicators UREA (**E**) and CREA (**F**). Data are presented as mean ± s.d. (*n* = 3 biologically independent samples). (**G**) H&E-stained digestive tissues (esophagus, small and large intestine) were analyzed at different time points after Ta-rCDs administration, with untreated controls included. Scale bar = 200 μm.

To evaluate the *in vivo* safety, rats were orally administered with the Ta-rCDs (5 mmol Ta/kg body weight) and monitored over a 14-day period for changes in body weight, blood biochemistry and tissue histopathology. The treatment group exhibited body weight profiles comparable to those of the control group, with no significant differences observed (Figure 6B). Blood biochemical analysis assessed liver and kidney function markers, including alanine aminotransferase (ALT), aspartate aminotransferase (AST), total protein (TP), albumin, creatinine (CREA), urea (UREA) and uric acid, all of which remained within normal physiological ranges, indicating minimal impact on hepatic and renal function (Figure 6C–F and Supplementary Figure S19). Moreover, histological analyses were performed on the GI tract (esophagus, small intestine and large intestine), major organs (heart, liver, spleen, lung and kidney) and ocular tissues (cornea, retina and optic nerve). H&E staining showed that all examined tissues remained histologically normal, with no signs of inflammation, necrosis or structural damage (Figure 6G and Supplementary Figures S20 and S21). The biodistribution of Ta following oral administration of Ta-rCDs was quantified in feces, urine and blood by ICP-OES at designated time points. Most of the administered Ta was recovered in feces within 48 h, whereas Ta levels in both blood and urine remained negligible throughout the observation period (Supplementary Figure S22). These findings indicated that orally administered Ta-rCDs were predominantly eliminated through fecal excretion with minimal systemic absorption. Collectively, these results demonstrated that Ta-rCDs were well tolerated *in vitro* and *in vivo*, confirming their safety and supporting the robustness of the imaging findings.

Compared with our previously reported Ta nanodots [44], Ta-rCDs substantially simplify both material synthesis and subsequent purification. The preparation of Ta nanodots involves multiple labor-intensive steps, including hydrothermal treatment with hydrofluoric acid, peroxide-citrate complexation, ethanol precipitation and freeze-drying, whereas Ta-rCDs are readily synthesized through a one-pot thermal carbonization of TaCl_5_, CA and urea followed only by dialysis and lyophilization. This streamlined procedure eliminates hydrofluoric acid and other complicated processing steps, making the synthesis safer and more amenable to large-scale production. In addition, the carbon dot framework provides excellent aqueous dispersibility and colloidal stability together with abundant surface functional groups, which not only simplify subsequent bioconjugation but also provide opportunities for developing targeted molecular imaging probes.

These advantages are particularly attractive for oral GI imaging. The ultrahigh solubility, excellent stability under physiological conditions and rapid fecal clearance with negligible systemic absorption minimize systemic exposure after oral administration while maintaining sufficient imaging performance throughout GI transit. Compared with BaSO_4_, which may cause peritoneal adhesions or granuloma formation following accidental extravasation, Ta-rCDs exhibited a more favorable safety profile and were directly compatible with conventional clinical X-ray and CT imaging systems. Although these characteristics support the translational potential of Ta-rCDs, further studies are required to establish good manufacturing practice-compliant production, evaluate the long-term safety of repeated or high-dose administration in larger animal models and assess cost-effectiveness before clinical application.

The current Ta-rCDs nevertheless remain passive CAs that primarily provide anatomical information, which limits their ability to detect early lesions or distinguish specific GI diseases. Their readily modifiable surface chemistry provides a feasible route to address this limitation through conjugation with disease-specific antibodies, peptides or other targeting ligands, thereby extending their capability from anatomical imaging toward molecular imaging of pathological processes such as tumor angiogenesis and inflammatory bowel disease. Future studies should focus on optimizing surface functionalization while maintaining colloidal stability and target specificity in the complex GI environment, which will be essential for expanding the utility of Ta-rCDs toward targeted molecular imaging.

## Conclusion

In conclusion, we show noninvasive diagnosis of diverse GI disorders using Ta-rCDs as an X-ray CA. Ta-rCDs were obtained through a facile and scalable high-temperature carbonization process, yielding a material with high Ta loading, gram-level production capability, good aqueous dispersibility, ultrasmall hydrodynamic size and strong X-ray attenuation performance. In models of small intestinal stricture and esophageal leakage, Ta-rCDs allowed both high-resolution CT and dynamic X-ray imaging, facilitating visualization of microlesions that are challenging to detect with conventional methods. Besides, Ta-rCDs can be efficiently metabolized following entry into the peritoneal cavity through GI leaks, preventing long-term accumulation. Additionally, comprehensive biosafety evaluations further confirmed their biocompatibility. In summary, our proposed Ta-rCDs provide a biocompatible and robust solution for noninvasive, real-time and high-sensitivity GI imaging, paving the way for the development of orally deliverable CAs suitable for clinical translation.

## Supplementary Material

rbag187_Supplementary_Data

## Data Availability

Data will be made available upon reasonable request.
